# Supplementary material for: Molecular Profiling of Axial Spondyloarthritis Patients Reveals an Association between Innate and Adaptive Cell Populations and Therapeutic Response to Tumor Necrosis Factor Inhibitors
Source: Biomolecules. 2024 Mar 21;14(3):382. doi: 10.3390/biom14030382 (PMC10967957; doi:10.3390/biom14030382)
Supplement: Supplementary file 1 [file biomolecules-14-00382-s001.zip › SuppMethods.pdf]

Molecular profiling of axial spondyloarthritis patients reveals an association between innate and adaptive cell populations and therapeutic response to TNFalpha inhibitors.

Supplementary Material

Criteria For Patients selection

Inclusion Criteria:

- r-axSpA according to Portuguese Society of Rheumatology (SPR) guidelines (1984 modified New York Criteria, but allowing the use of MRI as imaging criteria)
- Patient enrolment followed national guidelines for TNF antagonist use for the treatment of r-axSpA
- Adults between 18 to 75 years
- Ability to provide informed consent
- Corticosteroid therapy allowed (equivalent to ≤ 10 mg prednisone) and / or NSAID (nonsteroidal anti-inflammatory drug), stable dose in 4 weeks before study initiation
- Adequate contraception (barrier or hormonal) in men and women of childbearing age (patients and their partners
- Adequate renal and hepatic function (2 times ULN)

Exclusion Criteria:

- Current pregnancy or breastfeeding
- Previous treatment with biologic DMARD's (disease-modifying antirheumatic drug)
- Intraarticular (including sacroiliac joints) and periarticular injections within 28 days before screening.
- History of rheumatic disorder other than r-axSpA
- Other forms of spondylarthritis than r-axSpA
- Any uncontrolled medical condition (e.g., uncontrolled diabetes mellitus, unstable ischemic heart disease)
- History or signs of demyelinating disease
- Malignancy (except for completely treated squamous or basal cell carcinoma)
- Moderate to severe heart failure (NYHA class III/IV) Positive serology for hepatitis B, hepatitis C, or human immunodeficiency virus

- Active or latent tuberculosis (TB) or histoplasmosis or other severe infections such as sepsis, and opportunistic infections
- Infections requiring hospitalization or intravenous treatment with antibiotics within 30 days or oral treatment with antibiotics within 14 days before enrollment
- Ankylosis of the spine (syndesmophytes presence at all levels from D12 to S1 in X-ray (XR) lateral view)
- Hypersensitivity to the active substance or to any of the excipients

The ASAS20 was used as criteria to classify all patients as responders or non-responders, at week 14. With the ASAS outcome criteria, usually >60% reach ASAS 20 which means that we would need to include a larger number of patients to establish the subgroups for analysis. To have 80% power to detect a 0.5SD difference between groups at p=0.05 (paired t-test), we estimated that we would need samples from 18 responders and 18 non-responders. Thus, we included the number of patients necessary to ensure 18 non-responders, after which we closed the recruitment period.

Transcriptomics Analysis

RNA-Seq Library preparation and sequencing. Peripheral blood samples were collected into PAXgene Blood RNA System® tubes and stored at -80° C according to the manufacturer's recommendations[36]. Total RNA was extracted from whole blood samples according to the standard PAXgene protocol[33](Qiagen, 2008). The quantity of RNA was measured using a NanoDrop 2000/2000c Spectrophotometer according to the manufacturer's procedure (Thermo-Scientific, 2000); RNAs with a 260:280 ratio of ≥1.5 were sequenced as below. The quality and quantity of the libraries was assessed by Fragment Analyzer with the method of DNF-474-22 - HS NGS Fragment 1-6000bp (Agilent). Sequencing library preparation was performed using Illumina TruSeq stranded mRNA library preparation kits, with 100ng of total RNA as input. Libraries were sequenced on an Illumina NextSeq500 sequencer (average of 39 million reads per sample, 75 base-pair paired-end).

RNA-Seq data analysis: Raw sequencing reads were aligned to gencode (v32) transcripts using kallisto (version 0.46.1)[37], reaching an average of 86% reads assigned to genes (gene counts are in Supp. Table 11). The edgeR R package was used to normalize raw counts with the trimmed mean of M-values (TMM) normalization approach[38], and to filter low-expressed genes with the filterByExpr function. The limma R package was used to apply a voom transformation for variance stabilization[39], and to obtain differentially expressed genes through an empirical bayes approach. Genes were considered differentially expressed if the adjusted p-value of the test was less than 0.05. Functional enrichment analysis was performed using the fgsea R package, based on ranks of the moderated t-statistic from the empirical bayes. The per-gene variance explained by each variable was estimated using the variancePartition R package. Permutational multivariate analysis of variance (adonis) was performed using the vegan R package.

Inference of Immune cell populations from RNA-Seq data: Cibersort[40] was used to infer immune cell populations by comparing normalized log2(CPM) of the blood transcriptomes to the abbas et al. signatures[41]. To assess its accuracy, we correlated the relative frequencies obtained with RNA-Seq with values obtained with clinical hemograms (Supplemental Figure 8, pearson R=0.84 and p=3.3e-8 for Neutrophils, R=0.86 and p=1.2e-8 for Lymphocytes). Quantitative set analysis for gene expression (QUSAGE)[42] was used to assess the fold change of immune signature gene sets from Lewis et al.[43].

Proteomics

Immunoaffinity depletion of high-abundance proteins. Peripheral blood samples were collected into Clot Activator Tubes (Monovette Serum Gel Z- 7.5 mL, Sarsted) containing 100 µL of Protease Inhibitor Cocktail (Sigma-Aldrich). The six most abundant proteins in serum were depleted using the Multiple Affinity Removal Spin Cartridge Human 6 Kit (Agilent Technologies®) following manufacturer's instructions. The remaining proteins were concentrated using 4 mL Spin Concentrators with 5000 MWCO (Agilent Technologies®). A centrifugation was performed (with a fixed angle rotor) at 4000 x g and 10ºC until the sample reached a volume between 100 and 140 µL, after which it was recovered from the bottom of the concentrator pocket and stored at -20ºC until further analysis. In order to quantify the amount of protein in each sample, the QuantiPro™ BCA Kit (Sigma-Aldrich®) was used.

In-gel protein digestion. 50 µg of total proteins was diluted with MilliQ water to a final volume of 20 µL and 10 µL of LDS3X (Invitrogen™ by Life Technologies™) was added, for a final volume of 30 µL. Samples were heated for 5 min at 99ºC and briefly centrifuged (16,000 g for 1 min). The whole volume of the supernatant containing the soluble proteins was loaded on a NuPAGE 4-12% Bis-Tris (Invitrogen™ by Life Technologies™) gel and the proteins were subjected to SDS-PAGE electrophoresis for 5 min. After migration, the gels were stained with Coomassie SimplyBlue SafeStain (Invitrogen™ by Life Technologies™) for 5 min and washed with water overnight with gentle agitation. Polyacrylamide bands containing the stained proteome were cut by the limit of gel wells, between the front of migration and the well bottom. Each sample was treated and proteolyzed with trypsin Gold (Promega©) in presence of ProteaseMax detergent (Promega©) as previously described[44]. The final volume of peptide extract was 50 µL.

LC-MS/MS analysis. Tryptic peptides were analyzed with a Q-Exactive™ HF high resolution tandem mass spectrometer (ThermoFisher Scientific™) incorporating an ultra-high-field Orbitrap analyser as previously described[45]. Shortly, 10 µL of the resulting peptide mixtures for each sample were injected in a random order. First, peptides were desalted online on a reverse phase precolumn Acclaim PepMap 100 C18 (5 µm, 100 Å, 300 µm id x 5 mm), and then, they were resolved on a reverse phase column Acclaim PepMap 100 C18 (3 µm, 100 Å, 75 µm id x 500 mm) at a flow rate of 200 nL/min with a 90 min gradient of 4 to 25 % of B in 75 min and 25 to 40% of B in 15 min (being A: 0.1% HCOOH and B: 80% CH_3CN, 0.1% HCOOH). The Q-Exactive HF

instrument was operated according to a Top20 data-dependent method consisting in a scan cycle initiated with a full scan of peptide ions in the ultra-high-field Orbitrap analyzer, followed by serial selection of each of the 20 most abundant precursor ions, high energy collisional dissociation and MS/MS scans. Full scan mass spectra were acquired from *m/z* 350 to 1,500 with a resolution of 60,000. A peptide exclusion list was established for the most abundant immunodepleted proteins: serum albumin (https://www.uniprot.org/uniprot/P02768) complement C3 (P01024), alpha-2-macroglobulin (P01023), and apolipoprotein B-100 (P04114), in order to focus the analysis on the other proteins. Each MS/MS scan was acquired with a threshold intensity of 83.000, on potential charge states of 2+ and 3+ after ion selection performed with a dynamic exclusion of 10 sec, maximum Inclusion Time (IT) of 60 ms and an *m/z* isolation window of 2.0. MS/MS spectra at a resolution of 15.000 were searched using MASCOT 2.5.1 software (Matrix Science) against the Swissprot Human database downloaded in July 2019 (20.432 Homo sapiens protein sequences). The following parameters were used for MS/MS spectra assignation: full trypsin specificity, maximum of two missed cleavages, mass tolerances of 5 ppm on the parent ion and 0.02 Da on the secondary ions, fixed modification of carbamidomethyl cysteine (+57.0215), and oxidized methionine (+15.9949) and deamidated (NQ) (0.9840) as dynamic modifications.

Protein identification and relative quantification. After LC-MS/MS, a bioinformatic analysis was performed where all peptide matches with a MASCOT peptide score below a query identity threshold p-value of 0.05 were filtered and assigned to proteins. A total of 5.453.298 MS/MS spectra were recorded and 1.632.427 spectra were assigned to peptide sequences from the protein database – these peptide spectral matches are listed in Supp. Table 12. A protein identification was considered valid when at least two different non-ambiguous peptides were detected in the whole dataset. False discovery rate (FDR) for proteins was below 1% when applying these rules with the MASCOT decoy search mode. A total of 333 polypeptide sequences were identified based on at least 2 non-ambiguous peptides – from the initially 377 polypeptide sequences identified, 44 contaminant proteins (keratin and keratin associated proteins) were excluded from further analysis. For each validated protein (listed in Supp. Table 11), the number of MS/MS spectra for all detected non-ambiguous peptides or 'Spectral Count' (SC)[46] was used as a proxy of their abundances[47]. To further assess the value of SC as a measure of protein abundance, we compared clinically determined CRP levels with CRP levels measured by proteomics, and found these to be highly correlated (Supp. Fig. 9, pearson R=0.73, spearman rho=0.79, $p < 0.001$). Differential protein analysis was performed similarly to the transcriptomics, using the SC values as counts.

References:

36. Kruhøffer, M.; Dyrskjøt, L.; Voss, T.; Lindberg, R.L.; Wyrich, R.; Thykjaer, T.; Orntoft, T.F. Isolation of Microarray-Grade Total RNA, MicroRNA, and DNA from a Single PAXgene Blood RNA Tube. J. Mol. Diagn. 2007, 9, 452–458. https://doi.org/10.2353/jmoldx.2007.060175.
37. Bray, N.L.; Pimentel, H.; Melsted, P.; Pachter, L. Near-optimal probabilistic RNA-seq quantification. Nat. Biotechnol. 2016, 34, 525–527. https://doi.org/10.1038/nbt.3519.
38. Robinson, M.D.; Oshlack, A. A scaling normalization method for differential expression analysis of RNA-seq data. Genome Biol. 2010, 11, R25. https://doi.org/10.1186/gb-2010-11-3-r25.
39. Ritchie, M.E.; Phipson, B.; Wu, D.; Hu, Y.; Law, C.W.; Shi, W.; Smyth, G.K. Limma powers differential expression analyses for RNA-sequencing and microarray studies. Nucleic Acids Res. 2015, 43, e47. https://doi.org/10.1093/nar/gkv007.
40. Chen, B.; Khodadoust, M.S.; Liu, C.L.; Newman, A.M.; Alizadeh, A.A. Profiling Tumor Infiltrating Immune Cells with CIBERSORT. In em Cancer Systems Biology; von Stechow, L., Ed.; Springer: New York, NY, USA, 2018; Volume 1711, pp. 243–259. https://doi.org/10.1007/978-1-4939-7493-1_12.
41. Abbas, A.R.; Wolslegel, K.; Seshasayee, D.; Modrusan, Z.; Clark, H.F. Deconvolution of Blood Microarray Data Identifies Cellular Activation Patterns in Systemic Lupus Erythematosus. PLoS ONE 2009, 4, e6098. https://doi.org/10.1371/journal.pone.0006098.
42. Yaari, G.; Bolen, C.R.; Thakar, J.; Kleinstein, S.H. Quantitative set analysis for gene expression: A method to quantify gene set differential expression including gene-gene correlations. Nucleic Acids Res. 2013, 41, e170. https://doi.org/10.1093/nar/gkt660.
43. Lewis, M.J.; Barnes, M.R.; Blighe, K.; Goldmann, K.; Rana, S.; Hackney, J.A.; Ramamoorthi, N.; John, C.R.; Watson, D.S.; Kummerfeld, S.K.; et al. Molecular Portraits of Early Rheumatoid Arthritis Identify Clinical and Treatment Response Pheno-types. Cell Rep. 2019, 28, 2455–2470.e5. https://doi.org/10.1016/j.celrep.2019.07.091.
44. Hartmann, E.M.; Allain, F.; Gaillard, J.-C.; Pible, O.; Armengaud, J. Taking the Shortcut for High-Throughput Shotgun Pro-teomic Analysis of Bacteria. Methods Mol. Biol. 2014, 1197, 275–285. https://doi.org/10.1007/978-1-4939-1261-2_16.
45. Klein, G.; Mathé, C.; Biola-Clier, M.; Devineau, S.; Drouineau, E.; Hatem, E.; Marichal, L.; Alonso, B.; Gaillard, J.-C.; Lagniel, G.; et al. RNA-binding proteins are a major target of silica nanoparticles in cell extracts. Nanotoxicology 2016, 10, 1555–1564. https://doi.org/10.1080/17435390.2016.1244299.
46. Washburn, M.P.; Wolters, D.; Yates, J.R.R. Large-scale analysis of the yeast proteome by multidimensional protein identifica-tion technology. Nat. Biotechnol. 2001, 19, 242–247. https://doi.org/10.1038/85686.
47. Liu, H.; Sadygov, R.G.; Yates, J.R. A Model for Random Sampling and Estimation of Relative Protein Abundance in Shotgun Proteomics. Anal. Chem. 2004, 76, 4193–4201. https://doi.org/10.1021/ac0498563.

Supplementary Figure Legends

Supplemental Figure 1: The TNF inhibitor adalimumab induces a reduction in clinical disease parameters. A) Paired values of ASDAS-CRP scores, separated by response groups. Samples from the same patient are connected with a grey line between timepoints. **B)** Paired values of BASDAI scores, separated by response groups. In all cases, the differences were statistically significant ($p<0.05$), as estimated using a paired Wilcoxon signed rank test.

Supplemental Figure 2: Response to TNFi treatment is a significant factor distinguishing responders and non-responders at baseline. Sparse partial least squares discriminant analysis (sPLS-DA) of proteomics data in responders (AUC=1, p=1.1e-05) and non-responders (AUC=1, p=3.4e-05) using time as a variable of interest; In all cases, AUC and p-value correspond to the two best components of the sPLS-DA. In all graphs, ellipses represent 95% confidence intervals.

Supplemental Figure 3: Non-responders have fewer robustly differentially expressed genes/proteins between BL and W14. Volcano plot (log2 of the fold change versus -log10 of the unadjusted p-value) comparing the **A)** transcriptomics baseline samples versus week 14 samples in non-responders; for visual purposes, the p-values were plotted and not the adjusted p-values (FDR) as these were all 1 or very close to 1; **B)** proteomics baseline samples versus week 14 samples in non-responders; non-significant (NS) genes/proteins are in grey; in blue genes/proteins that are statistically significant but have a mild fold change (less than 2); some of the blue genes names are displayed in the plot.

Supplemental Figure 4: TNFi treatment in non-responders acts in the same pathways as responders. A) Barplot displaying the Normalized Enrichment Score (NES) of representative significant pathways resulting from a gene set enrichment analysis (GSEA) comparing transcript expression of week 14 (W14) against baseline (BL) non-responder samples.

Supplemental Figure 5: Transcriptomic and proteomic differences detected between BL and W14 in responders are attenuated between responders and non-responders at W14. Box plot of the log2 fold change of genes: **A)** "R: W14 vs BL" are w14 samples against baseline samples in responders; "W14: R vs NR" are responder versus non-responders at w14, Only the genes differentially expressed from "R: W14 vs BL" are represented. **B)** Same as A but regarding non-responders. **C)** Same as A but regarding proteins. **D)** Same as A but regarding proteins in non-responders. In all cases $p<0.05$.

Supplemental Figure 6: Markers of inflammation are already lowered in the plasma after 3-5 days of adalimumab treatment, in both responders and non-responders. A) Mean clinical CRP values in different time points, in responders and non-responders; Mean sCPM values in different time points, in responders and non-responders in **B)** CRP; **C)** HP; **D)** APOA2. Error bars represent standard deviation.

Supplemental Figure 7: Blood transcriptome data at baseline suggests that response to adalimumab derives from an interplay between innate and adaptive immunity. A) Heatmap representation of fold change values from a quantitative set analysis for gene expression (QUSAGE) used with immune signature gene sets from Lewis et al. W14.R_BL.R indicates comparison of week 14 samples against baseline samples in responders; W14.NR_BL.NR indicates comparison of week 14 samples against baseline samples in non-responders; BL.R_BL.NR indicates comparison of responders against non-responders at baseline; W14.R_W14.NR indicates comparison of responders against non-responders at week 14 (* indicates p-value of fold-change < 0.05; ** p-value < 0.01; *** p-value < 0.001); **B)** Heatmap representation of a cross-correlation analysis of the relative frequencies of different immune populations and clinical parameters; only significant correlations are displayed. Highly correlated variables are clustered together.

Supplemental Figure 8: RNA-Seq derived estimates of white blood cell populations correlate well with values from clinical hemograms. A) Correlation between Lymphocyte frequencies derived from RNA-Seq or from clinical hemograms (Pearson R=0.86, p=1.16e-8, n=27) **B)** Correlation between Neutrophil frequencies derived from RNA-Seq or from clinical hemograms (Pearson R=0.84, p<0.001, n=27) **C)** Ratio between Neutrophil and Lymphocytes derived from hemograms at baseline (Wilcoxon rank-sum test p=0.006, responders n=10, non-responders n=8) **D)** Ratio between Neutrophil and Lymphocytes derived from hemograms at week 14 (Wilcoxon rank-sum test p=0.79, responders n=7, non-responders n=4). **E)** Ratio between Neutrophil and Lymphocytes derived from Cibersort estimates at baseline (Wilcoxon rank-sum test p=0.008, responders n=16, non-responders n=17) **F)** Ratio between Neutrophil and Lymphocytes derived from Cibersort estimates at week 14 (Wilcoxon rank-sum test p=0.78, responders n=18, non-responders n=17). Colors represent response group and time point.

Supplemental Figure 9: Clinical CRP measures correlate well with CRP expression measurements from the proteomics assay. Correlation between clinical CRP and the sCPM values for CRP in the proteomics data. Spearman rho = 0.79; Pearson correlation on the log scale = 0.72; in both cases p-value<0.001. Values were log transformed, as the correlation seemed non-linear (the spearman correlation is the same as in the linear scale, and pearson correlation is still 0.64, p<0.001).

Supplementary Tables

Supplementary Table 1. **Results of the differential expression analysis comparing the transcriptomics of responders between week 14 and baseline.** For each gene we indicate the estimated log2(Fold Change), average expression (across all samples), moderated t-statistic, p-value, adjusted p-value, and B-statistic (posterior log-odds of differential expression). Genes are presented ordered by the adjusted p-value.

Supplementary Table 2. Results of the differential expression analysis comparing the proteomics of responders between week 14 and baseline. Same as Supplementary Table 1, but for proteomics data.

Supplementary Table 3. Results of the differential expression analysis comparing the transcriptomics of non-responders between week 14 and baseline. Same as Supplementary Table 1, but for non-responders.

Supplementary Table 4. Results of the differential expression analysis comparing the proteomics of non-responders between week 14 and baseline. Same as Supplementary Table 2, but for non-responders.

Supplementary Table 5. Results of the differential expression analysis comparing the proteomics of responders between 3-5 days and baseline. Same as Supplementary Table 2, but comparing time-point 2 (3-5 days) with baseline, in responders.

Supplementary Table 6. Results of the differential expression analysis comparing the proteomics of responders between 2 weeks and baseline. Same as Supplementary Table 2, but comparing time-point 3 (2 weeks) with baseline, in responders.

Supplementary Table 7. Results of the differential expression analysis comparing the proteomics of non-responders between 3-5 days and baseline. Same as Supplementary Table 2, but comparing time-point 2 (3-5 days) with baseline, in non-responders.

Supplementary Table 8. Results of the differential expression analysis comparing the proteomics of non-responders between 2 weeks and baseline. Same as Supplementary Table 2, but comparing time-point 3 (2 weeks) with baseline, in non-responders.

Supplementary Table 9. Results of the differential expression analysis comparing the proteomics at baseline between responders and non-responders. Same as Supplementary Table 2, but comparing responders with non-responders at baseline.

Supplementary Table 10. Results of the differential expression analysis comparing the transcriptomics at baseline between responders and non-responders. Same as Supplementary Table 1, but comparing responders with non-responders at baseline.

Supplementary Table 11. RNA-Seq gene counts from kallisto.

Supplementary Table 12. List of proteins identified by proteomics and their abundances estimated by spectral counts.
