# Supplementary figures and images for: Molecular Profiling of Axial Spondyloarthritis Patients Reveals an Association between Innate and Adaptive Cell Populations and Therapeutic Response to Tumor Necrosis Factor Inhibitors
Source: Biomolecules. 2024 Mar 21;14(3):382. doi: 10.3390/biom14030382 (PMC10967957; doi:10.3390/biom14030382)

Supplemental Figure 1

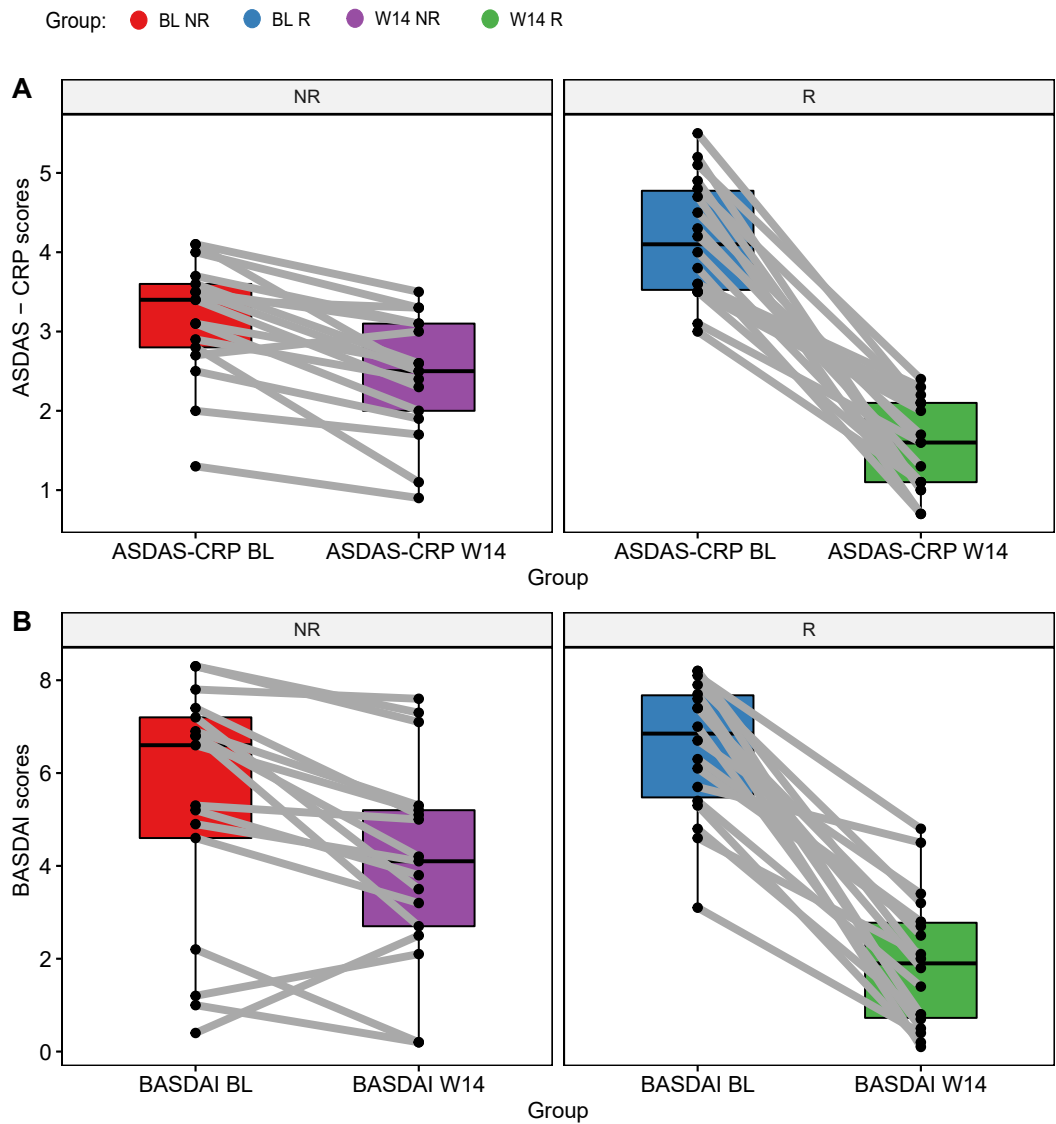

Supplement: Supplementary file 1 [file biomolecules-14-00382-s001.zip › SuppFig1.pdf]

Supplemental Figure 2

Group: BL NR BL R W14 NR W14 R

Proteomics: Baseline versus Week 14

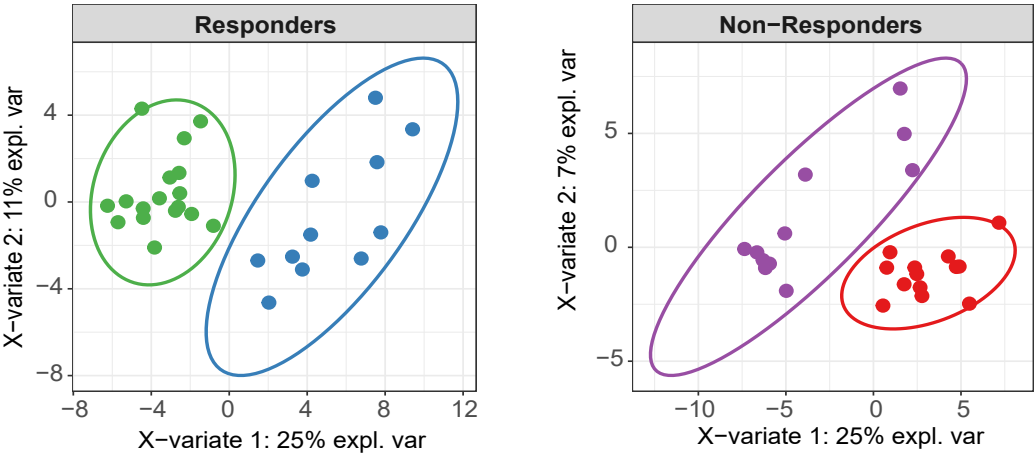

Supplement: Supplementary file 1 [file biomolecules-14-00382-s001.zip › SuppFig2.pdf]

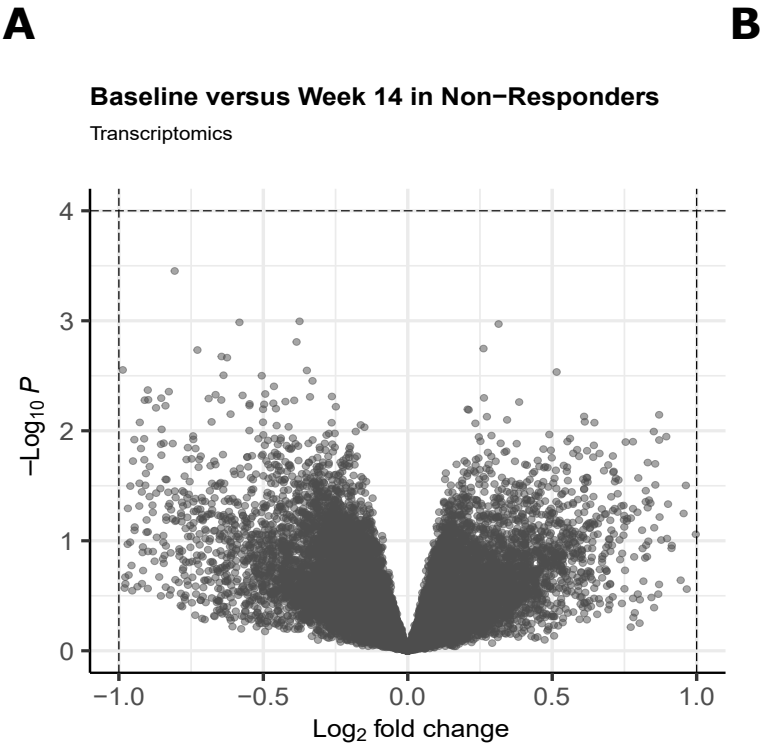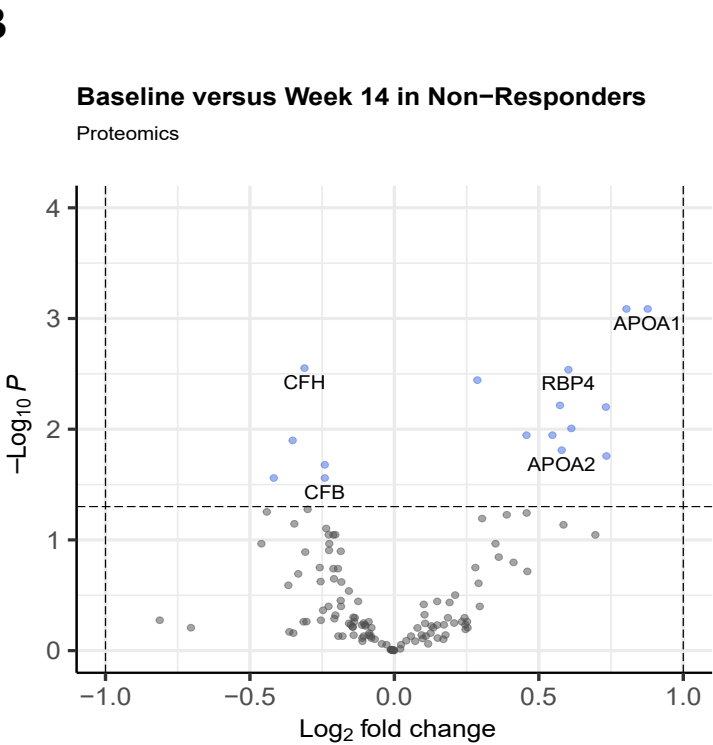

Supplement: Supplementary file 1 [file biomolecules-14-00382-s001.zip › SuppFig3.pdf]

Supplemental Figure 4

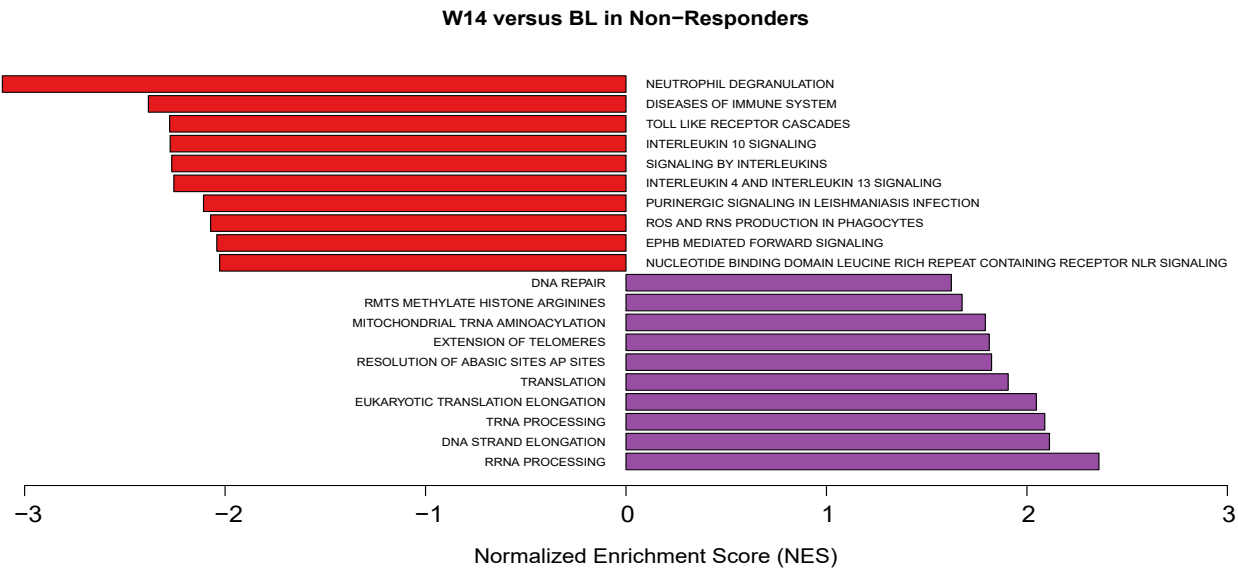

Supplement: Supplementary file 1 [file biomolecules-14-00382-s001.zip › SuppFig4.pdf]

Supplemental Figure 5

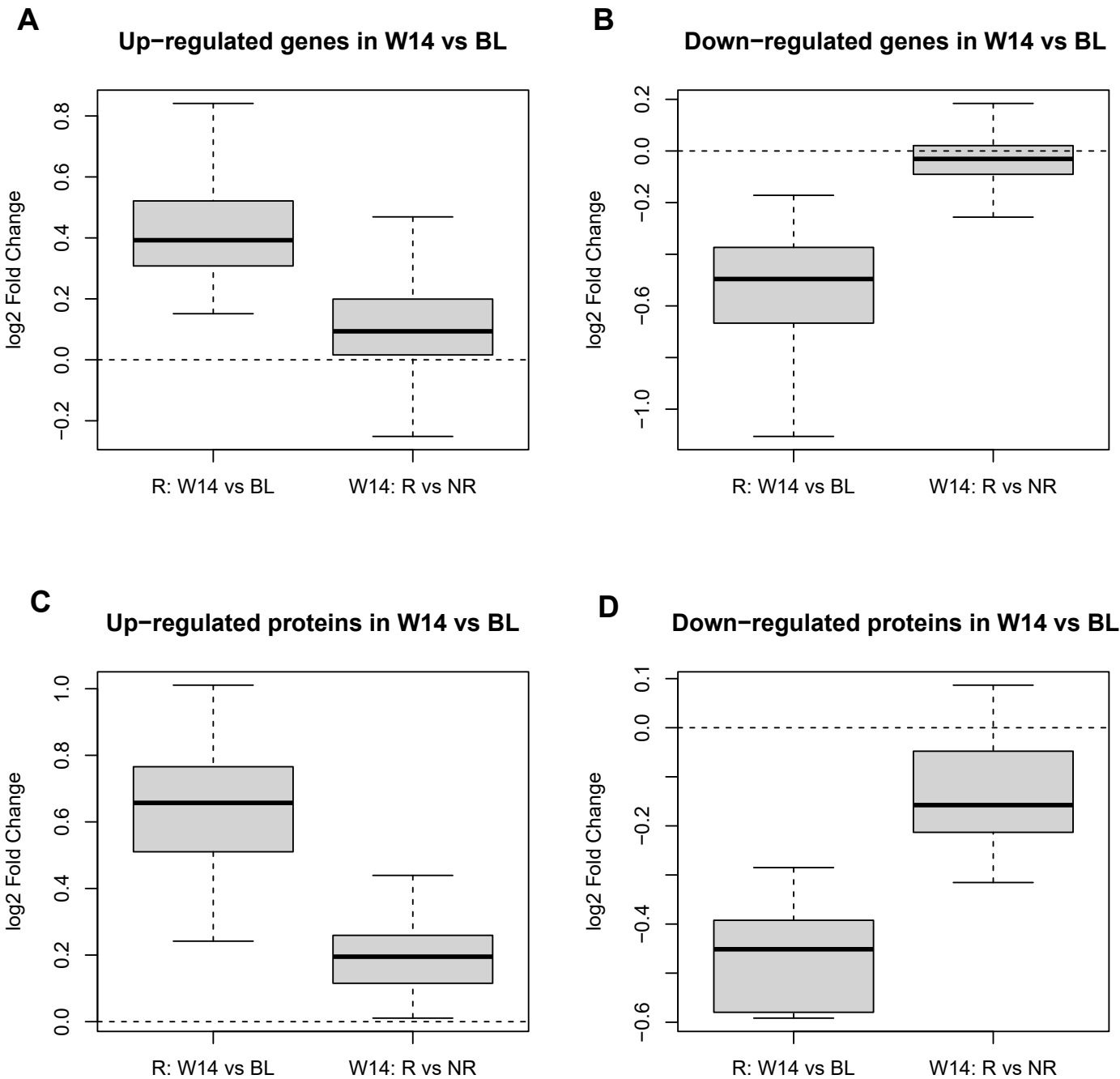

Supplement: Supplementary file 1 [file biomolecules-14-00382-s001.zip › SuppFig5.pdf]

Supplemental Figure 6

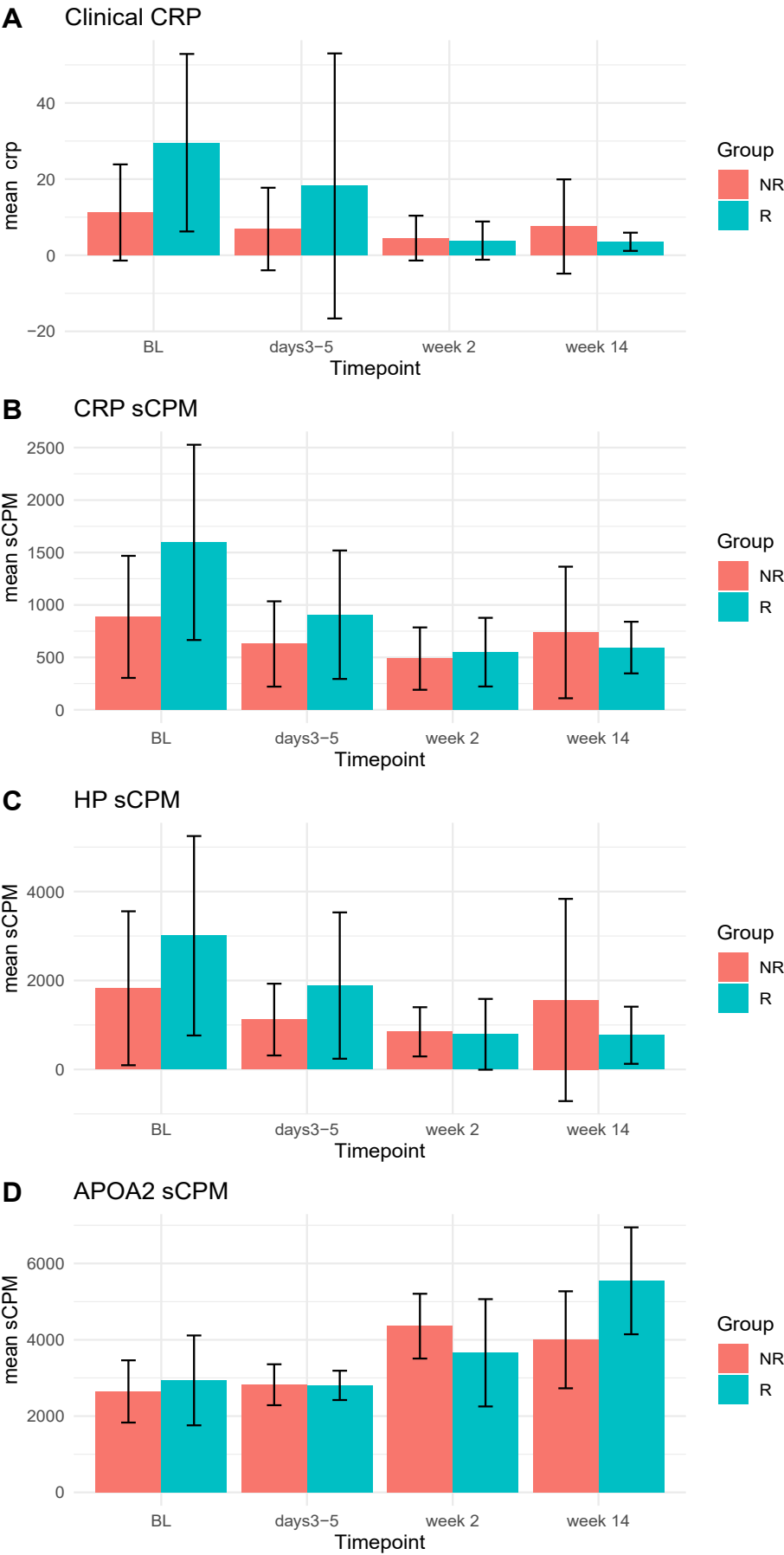

Supplement: Supplementary file 1 [file biomolecules-14-00382-s001.zip › SuppFig6.pdf]

Supplemental Figure 7

A

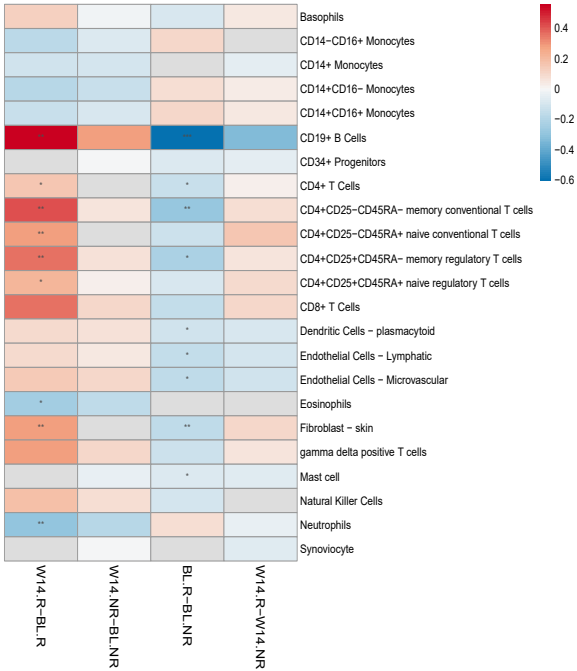

B

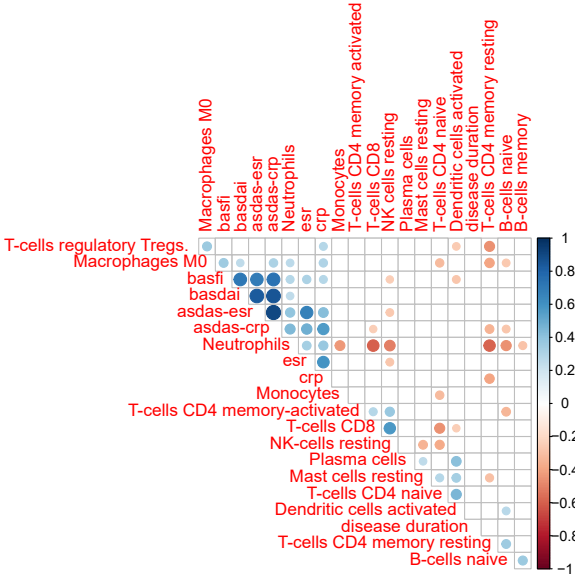

Supplement: Supplementary file 1 [file biomolecules-14-00382-s001.zip › SuppFig7.pdf]

Supplemental Figure 8

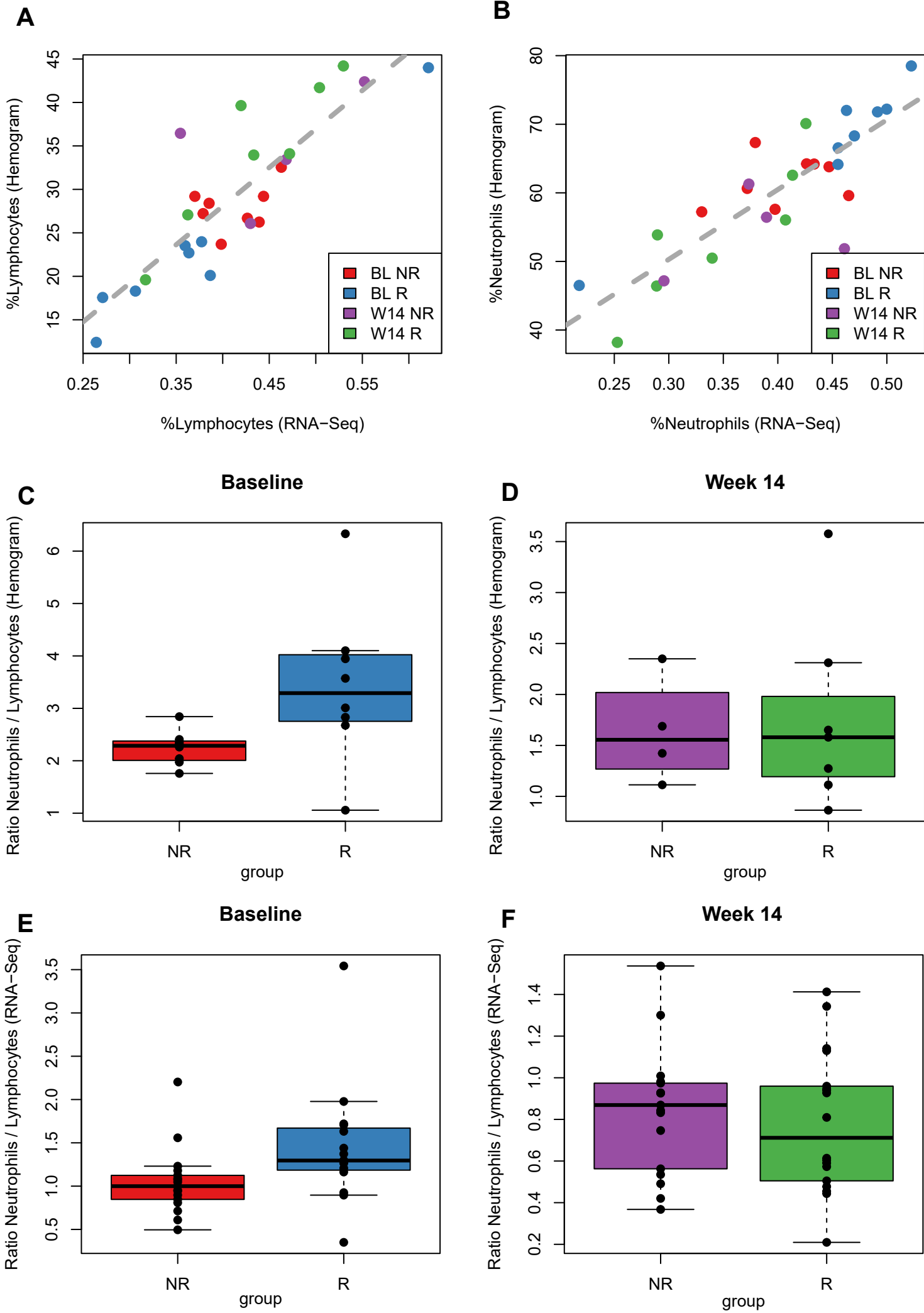

Supplement: Supplementary file 1 [file biomolecules-14-00382-s001.zip › SuppFig8.pdf]

Supplemental Figure 9

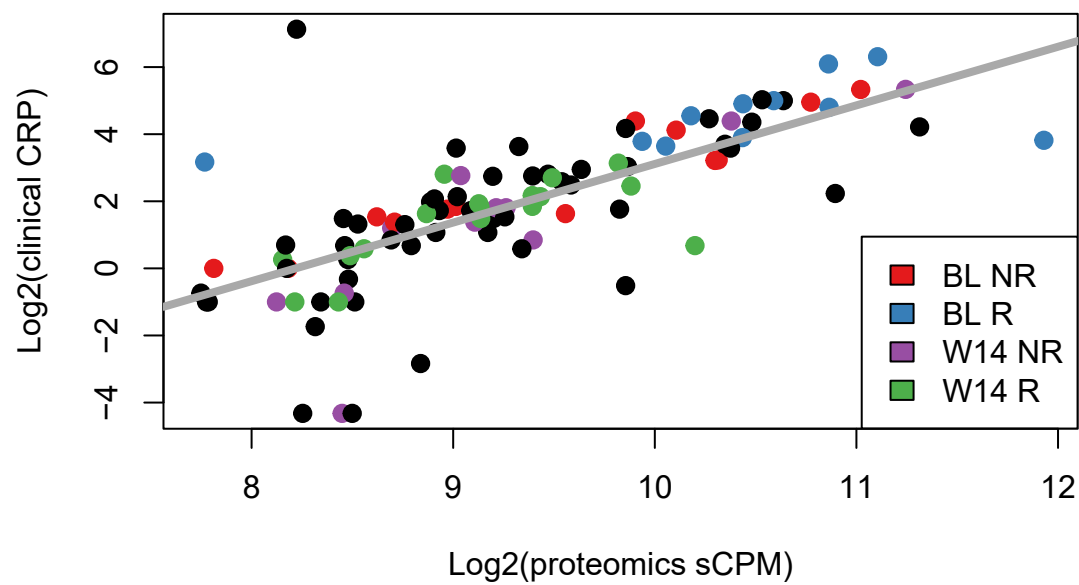

Supplement: Supplementary file 1 [file biomolecules-14-00382-s001.zip › SuppFig9.pdf]
